# Use of Patient Preference Information in Benefit–Risk Assessment, Health Technology Assessment, and Pricing and Reimbursement Decisions: A Systematic Literature Review of Attempts and Initiatives

**DOI:** 10.3389/fmed.2020.543046

**Published:** 2020-10-26

**Authors:** Lylia Chachoua, Monique Dabbous, Clément François, Claude Dussart, Samuel Aballéa, Mondher Toumi

**Affiliations:** ^1^Laboratory EA 3279 – CEReSS, Aix-Marseille University, Life Sciences and Health Department of Clinical Research and Public Health, Marseille, France; ^2^Creativ-Ceutical, Paris, France; ^3^University Lyon 1, Lyon, France

**Keywords:** preference measurement, decision-making, health technology assessment, benefit-risk assessment, patient preference

## Abstract

**Objectives:** Inclusion of patient preference (PP) data in decision making has been largely discussed in recent years. Healthcare decision makers—regulatory and health technology assessment (HTA)—are more and more conscious of the need for a patient-centered approach to decide on optimal allocation of scarce money, time, and technological resources. This literature review aims to examine the use of and recommendations for the integration of PP in decision making.

**Methods:** A literature search was conducted through PubMed/Medline in May 2019 to identify publications on PP studies used to inform benefit–risk assessments (BRAs) and HTAs and patient-centered projects and guidelines related to the inclusion of PPs in health policy decision making. After title and abstract screening and full-text review, selected publications were analyzed to retrieve data related to the collection, use, and/or submission of PPs informing BRA or HTA as well as attempts and initiatives in recommendations for PPs integration in decision-making processes.

**Results:** Forty-nine articles were included: 24 attempts and pilot project discussions and 25 PP elicitation studies. Quantitative approaches, particularly discrete choice experiments, were the most used (24 quantitative elicitation studies and 1 qualitative study). The objective of assessing PPs was to prioritize outcome-specific information, to value important treatment characteristics, to provide patient-focused benefit–risk trade-offs, and to appraise the patients' willingness to pay for new technologies. Moreover, attempts and pilot projects to integrate PPs in BRAs and HTAs were identified at the European level and across countries, but no clear recommendations have been issued yet. No less than seven public and/or private initiatives have been undertaken by governmental agencies and independent organizations to set guidance targeting improvement of patients' involvement in decision making.

**Conclusion:** Despite the initiatives undertaken, the pace of progress remains slow. The use of PPs remains poorly implemented, and evidence of proper use of these data in decision making is lacking. Guidelines and recommendations formalizing the purpose of collecting PPs, what methodology should be adopted and how, and who should be responsible for generating these data throughout the decision-making processes are needed to improve and empower integration of PPs in BRA and HTA.

## Introduction

Patients are the most familiar with their own health conditions. They are best positioned to provide a real-world understanding of their experiences and define their treatment preferences based on benefits and harms of treatment outcomes (1–9). As a result, an extensive trend toward making more patient-centric healthcare decisions has emerged, and experts have considered three possible levels of patient involvement referred to as micro, meso, and macro levels reliant on whether the involvement impacts patient–physician day-to-day interactions, a specific disease area, or resource allocation and healthcare policy decisions, respectively (10). While the role of patient with regard to micro-level decision, i.e., shared decision making, has significantly evolved in the last decades, when it comes to health authorities' decision making, the relative importance of patient voice remained unclear. Recently, health technology assessment (HTA) organizations, regulatory agencies, and decision-making bodies largely have started to explore opportunities for incorporation of patients' perspective in their decisions (1, 3, 11, 12). Furthermore, patients are claiming this greater role in healthcare decision making (3, 5, 12, 13).

In response to the growing interest of patient involvement, two trends, not mutually exclusive, have emerged: the first trend is “direct involvement” of patients in decision making, which is in favor of patients participating in discussions, such as through committees, advisory groups, or just testimonies, while the second trend is “indirect patient involvement” through studies allowing for the assessment of patient preferences (PPs) in a more systematic way (7, 10, 13). Moreover, researchers and policymakers have developed tools to assess PPs and initiated a variety of efforts and attempts to better include these PPs in benefit–risk assessment (BRA) and HTA decisions. As it still remains unclear how patient voices may formally be included in decision making and how rigorous patient evidence must be for acceptance by these decision makers (2, 12, 14–17), the primary objective of this review is to clarify the PP elicitation methodology and to examine the use of and available guidance for the integration of PPs in decision making.

## Methods

### Search Strategy

A literature search was performed on PubMed/Medline on May 2019. The search strategy detailed in Table 1 aimed to identify publications on PPs used to inform BRAs and HTAs. All relevant terms related to concepts of interest were included such as “patient preference,” “preference measurement methods and tools,” “decision-making process,” “benefit–risk assessment,” “health technology assessment,” and “pricing and reimbursement.” A supplementary hand search of references from included references was conducted.

**Table 1:** Search strategy.

**#**	**Concept**	**Query**	**Results**
S1	Patient preferences	(((((((((((((“patient preference” OR “patient preference/choice”))) OR “patients preferences”) OR “patient preferences”) OR “patients preference”) OR ((“patient input” OR “patient inputs”))) OR “patients input”) OR “patient choice”) OR ((“patients choice” OR “patients choices”))) OR “patient choices”) OR “patient perspective”) OR ((“patients perspective” OR “patients perspectives”))) OR “patient perspectives”	23,208
S2	Preference measurement methods and tools	((((((((((((((((“interview”) OR “survey”) OR ((“focus group” OR “focus group/consultation” OR “focus group/interview” OR “focus group/interview data” OR “focus group/interview questions” OR “focus group/interview transcripts” OR “focus group/interviews”))) OR “patient panel”) OR “preference measurement”) OR “preference elicitation”) OR ((“preference measure” OR “preference measurements” OR “preference measures”))) OR ((“preference rate” OR “preference rates” OR “preference rating” OR “preference ratings”))) OR ((“preference rank” OR “preference ranking” OR “preference rankings” OR “preference ranks”))) OR ((“pairwise comparison” OR “pairwise comparison experiment” OR “pairwise comparison method” OR “pairwise comparison methods” OR “pairwise comparison procedure” OR “pairwise comparison survey” OR “pairwise comparison test” OR “pairwise comparison testing” OR “pairwise comparison tests”))) OR “choice based”) OR ((“time trade off” OR “time trade off elicitation” OR “time trade off preference” OR “time trade off preferences”))) OR “discrete choice”) OR ((“standard gamble” OR “standard gamble preference”))) OR “swing weighting”) OR “best worst”) OR “contingent valuation”	639,175
S3	Decision making	(((“decision making”) OR “multicriteria decision”) OR “multi criteria decision”) OR “mcda”	191,923
S4	Medicinal product life cycle phase	((((((((((((“benefit risk” OR “benefit risk assessment” OR “benefit risk assessment method” OR “benefit risk assessment methods” OR “benefit risk assessments” OR “benefit risk preferences”))) OR “health technology/technology assessment”) OR “biomedical technology assessment”) OR “hta”) OR “reimbursement”) OR ((“reimbursement decision” OR “reimbursement decision making” OR “reimbursement decisions”))) OR((“pricing” OR “pricing/reimbursement” OR “pricing and reimbursement”))))) OR ((“european medicine agency” OR “european medicines agency” OR “european medicines agency ema”))) OR ((“food and drug administration” OR “food and drug administration fda”))	127,178
S5		S3 OR S4	314,637
S6		S1 AND S2 AND S5	1,246
S7	Excluded concept	“Shared decision making”	6,749
S8		S6 NOT S7	983

### Selection of Studies

The titles and abstracts of all the citations identified by the search strategy were independently screened for eligibility by two reviewers. After reaching a consensus, full texts of selected abstracts were screened. Included articles were reviewed for data extraction.

Studies published in English, which reported PP elicitation and initiatives of PP implementation in healthcare decision making, were considered. Included studies were either primary research studies that prospectively collected PPs or specific patient-centered projects and guidelines related to the inclusion of PPs in decision making. Studies assessing PPs for shared decision making, i.e., the process in which clinicians and patients work together to make decisions and select tests, treatments, and care plans (18), were excluded as not directly linked to heath policy decision making. No start date was specified so that all studies published up through end of May 2019 would be included. No restriction was set regarding the geographical scope.

### Assessment of Risk of Bias

As the aim of research was to assess to what extent PPs are used in policy decision making and how influential they are, the authors did not report the outcomes and the primary results of each individual PP study included. As such, the authors did not evaluate sources of bias in each individual study.

### Data Extraction and Synthesis

For each publication included, data extracted included the type of article, the region studied, the type of elicitation method used, the disease area, and the decision-making process engaged. Any relevant data related to the collection, use, or submission of PPs informing BRAs, HTAs, or drug pricing were retrieved. Attempts and initiatives at setting recommendations for the integration of PPs in the decision-making processes were also identified and classified by the decision-making process as well as institutional level engaged and geographical scope.

## Results

**Figure 1:**
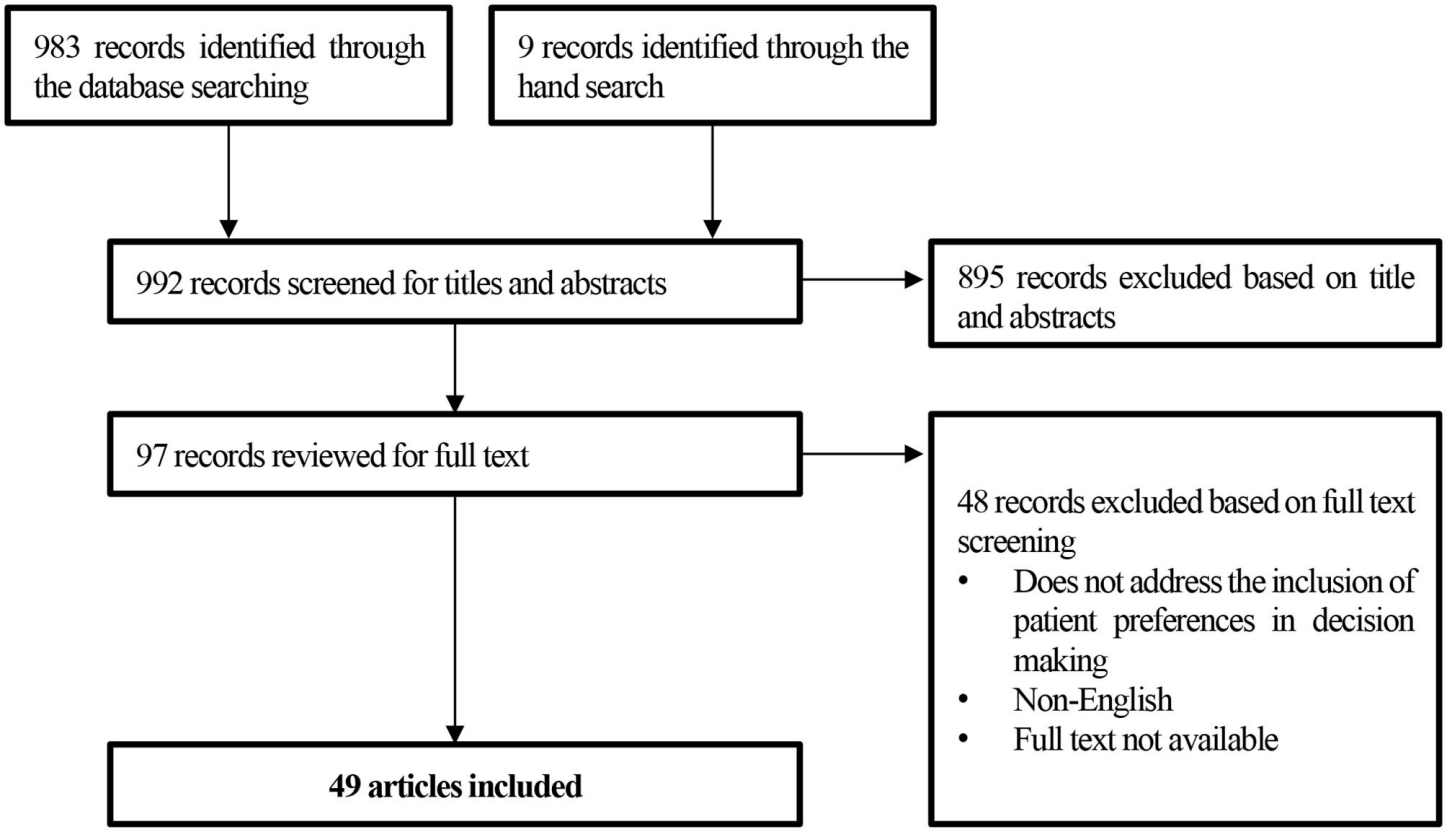
PRISMA flow diagram for study selection.

**Figure 2:**
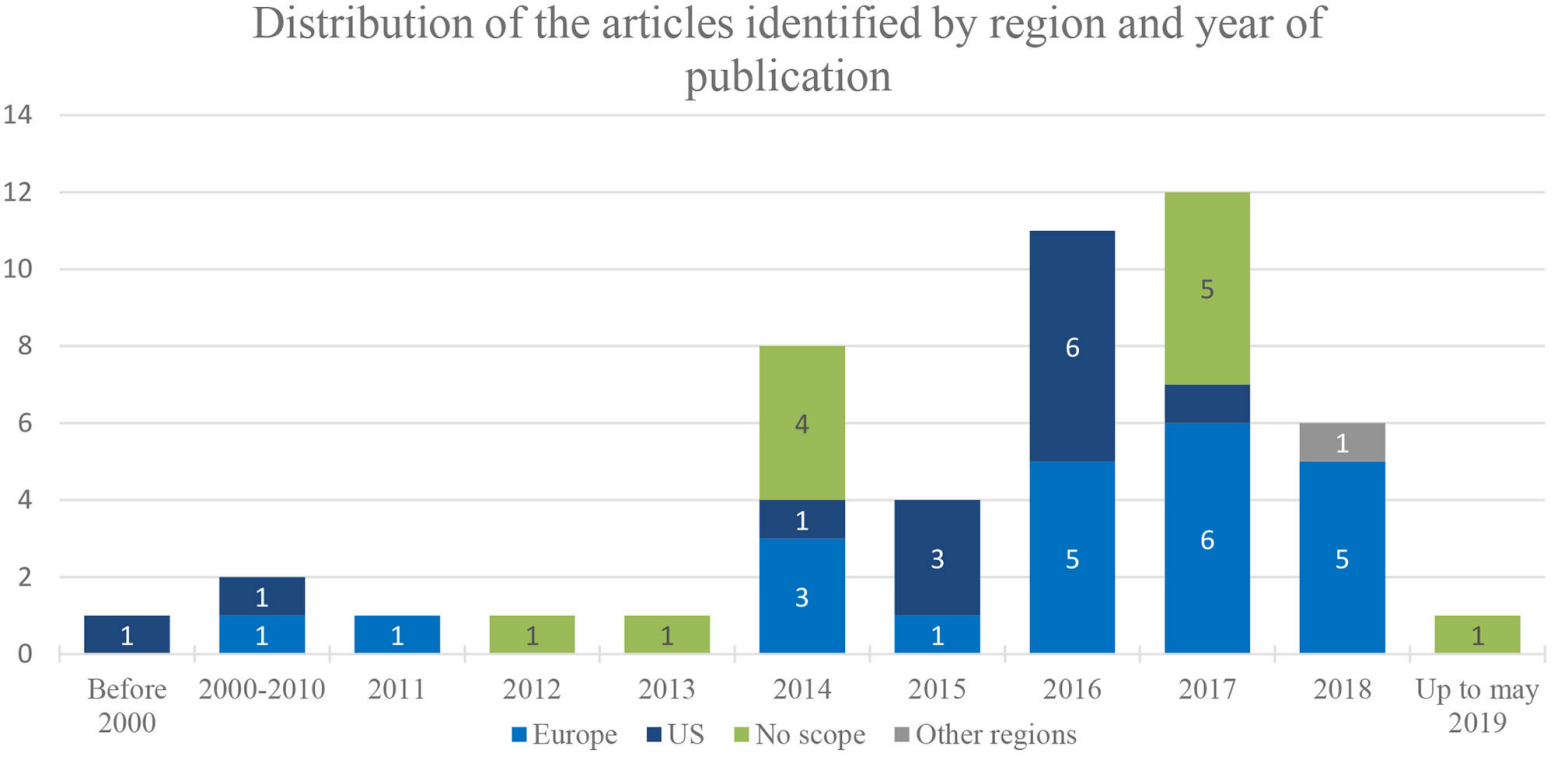
Distribution of the articles identified by region and year of publication.

Based on the search strategy and the supplementary hand search, 992 publications were identified (Figure 1). In total, 49 publications met all the criteria for inclusion in the review: 24 attempts and pilot projects discussions and 25 PP elicitation studies. The majority of the research and efforts identified through the literature review were recent initiatives, dated since 2014 (*n* = 42), while the earliest article considering PPs in decision making was published in 1999 (19). Moreover, most of the articles focused on European and US studies, efforts, and attempts. Only one initiative was undertaken out of Europe and the USA, in Australia (20) (Figure 2).

### PP Studies and Elicitation Methods

Of the 49 publications included, 25 records were studies eliciting PPs informing either BRAs (*n* = 15) and/or HTAs (*n* = 10) of medicinal technologies. Among them, six studies aimed also at informing pricing and/or reimbursement properly (Table 2).

**Table 2:** Type and methodology of PP studies identified in the SLR.

**First author (year)**	**Region/country**	**MPLC phases in which applications were identified**	**Methods used**		**Disease area**	**Attribute**	**References**
			**Qualitative**	**Quantitative**			
Vennedey et al. (2018)	Europe (Germany)	HTA, Reimbursement	-	DCE	Periodontal disease	Cost, Convenience, QoL	(11)
Holmes et al. (2018)	Europe (UK)	BRA	-	DCE	Epilepsy	Efficacy, Safety, QoL	(21)
Fifer et al. (2018)	Australia	HTA, Pricing/Reimbursement	-	DCE	Diabetes	Efficacy, Safety, Cost, Convenience	(20)
Postmus et al. (2018)	Europe (UK)	BRA	-	DCE	Multiple myeloma	Efficacy, Safety	(22)
Milovanovic et al. (2017)	Europe (Italy)	HTA	Survey/Questionnaire	-	Anemia/inflammatory bowel disease	Efficacy, Safety Convenience	(23)
Von Arx et al. (2016)	Europe (Denmark)	BRA	-	DCE	Diabetes	Efficacy, Safety	(24)
Eliasson et al. (2017)	Europe (France, Germany, UK)	BRA	-	DCE	Oncology	Efficacy, Safety, Convenience, QoL	(25)
Eliasson et al. (2017)	Europe (UK)	BRA, HTA	-	DCE	Psoriasis	Efficacy, Safety, Cost, Convenience	(26)
Muhlbacher et al. (2017)	Europe (Germany)	HTA	-	DCE	Hepatitis C	Efficacy, Safety, Cost, Convenience	(27)
Janssen et al. (2016)	Europe (Germany)	HTA	-	VAS	Kidney diseases	Efficacy, Safety, Convenience, QoL	(16)
Hollin et al. (2016)	USA	BRA	-	BWS	Duchene muscular dystrophy	Efficacy, Safety, QoL	(28)
Hollin et al. (2017)	USA	BRA	-	BWS + DCE	Duchene muscular dystrophy	Efficacy, Safety, Convenience	(29)
Morel et al. (2016)	Europe (UK)	BRA	-	DCE	Rare diseases	Efficacy, Safety, Convenience, QoL	(14)
Hauber et al. (2016)	USA	BRA	-	DCE	Eczema	Efficacy, Safety	(30)
Janssen et al. (2016)	USA	BRA, Pricing	-	BWS + DCE	Diabetes	Efficacy, Safety, Cost, Convenience	(31)
Muhlbacher et al. (2016)	Europe (Germany)	HTA	-	DCE	Hepatitis C	Efficacy, Safety, Convenience	(32)
Postmus et al. (2015)	Europe	BRA	-	MCDA	Oncology	Efficacy, Safety	(33)
Roy et al. (2015)	USA	HTA, Pricing	-	DCE	Insomnia	Efficacy, Safety, Cost, Convenience	(34)
Ho et al. (2015)	USA	BRA	-	DCE	Obesity	Efficacy, Safety, Convenience	(35)
Mol et al. (2014)	Europe (Netherlands)	BRA	-	DCE	Diabetes	Efficacy, Safety	(36)
Peay et al. (2014)	USA	BRA	-	BWS	Duchene muscular dystrophy	Efficacy, Safety	(37)
Danner et al. (2011)	Europe (Germany)	HTA	-	AHP	Depression	Efficacy, Safety, QoL	(8)
Johnson et al. (2010)	USA	BRA	-	DCE	Irritable bowel syndrome	Efficacy, Safety	(38)
Aristides et al. (2004)	Europe (France, Germany, Italy, Spain, UK)	HTA, Pricing/Reimbursement	-	DCE	Diabetes	Efficacy, Safety, Cost, Convenience	(39)
Sorum (1999)	US	Pricing	-	Rating scale + SG	ORL/infection	Efficacy, Safety, Cost	(19)

*BRA, benefit–risk assessment; HTA, health technology assessment; MPLC, medicinal product life cycle; DCE, discrete choice experiment; VAS, visual analog scale; BWS, best–worst scaling; AHP, analytic hierarchy process; SG, standard gamble; MCDA, multicriteria decision analysis; QoL, quality of life*.

Attributes assessed were predominantly efficacy and safety (*n* = 24) and treatment convenience such as mode and frequency of administration (*n* = 14). PPs regarding treatment cost (*n* = 8) and health-related quality of life (*n* = 7) were less frequently elicited.

Among disease areas of interests, metabolic disorders (*n* = 6) such as diabetes and obesity and rare diseases (*n* = 4) including Duchenne muscular dystrophy were the most frequently evaluated. Oncology including multiple myeloma (*n* = 3), central nervous system disorders (epilepsy insomnia and depression) (*n* = 3), and infectious diseases such as hepatitis C and otitis (*n* = 3) were less frequently elicited. Remaining areas such as autoimmune disorders (psoriasis and eczema), gastrointestinal troubles (inflammatory bowel disorders and irritable bowel syndrome), and periodontal and kidney disorders were barely assessed (*n* = 2, *n* = 2, *n* = 1, and *n* = 1, respectively).

Moreover, several PP elicitation methods and tools have been identified throughout the review. Only one qualitative method eliciting PPs among patients with inflammatory bowel disease in Italy has been retrieved (23). A higher trend toward using quantitative tools to elicit PPs was identified (*n* = 24). For instance, two-thirds of studies included in the literature review have been developed using a choice-based tool, either a discrete choice experiment (DCE) (*n* = 18) or a best–worst scaling (BWS) method used alone or associated with a DCE (*n* = 4). Multicriteria decision analysis (MCDA) approaches (*n* = 2), visual analog scales (*n* = 1), rating scales (*n* = 1), and standard gambles (*n* = 1) were less commonly used in PP studies aiming at informing BRAs, HTAs, and pricing and reimbursement decisions.

### Attempts and Initiatives for Integration of PPs in Decision Making

**Figure 3:**
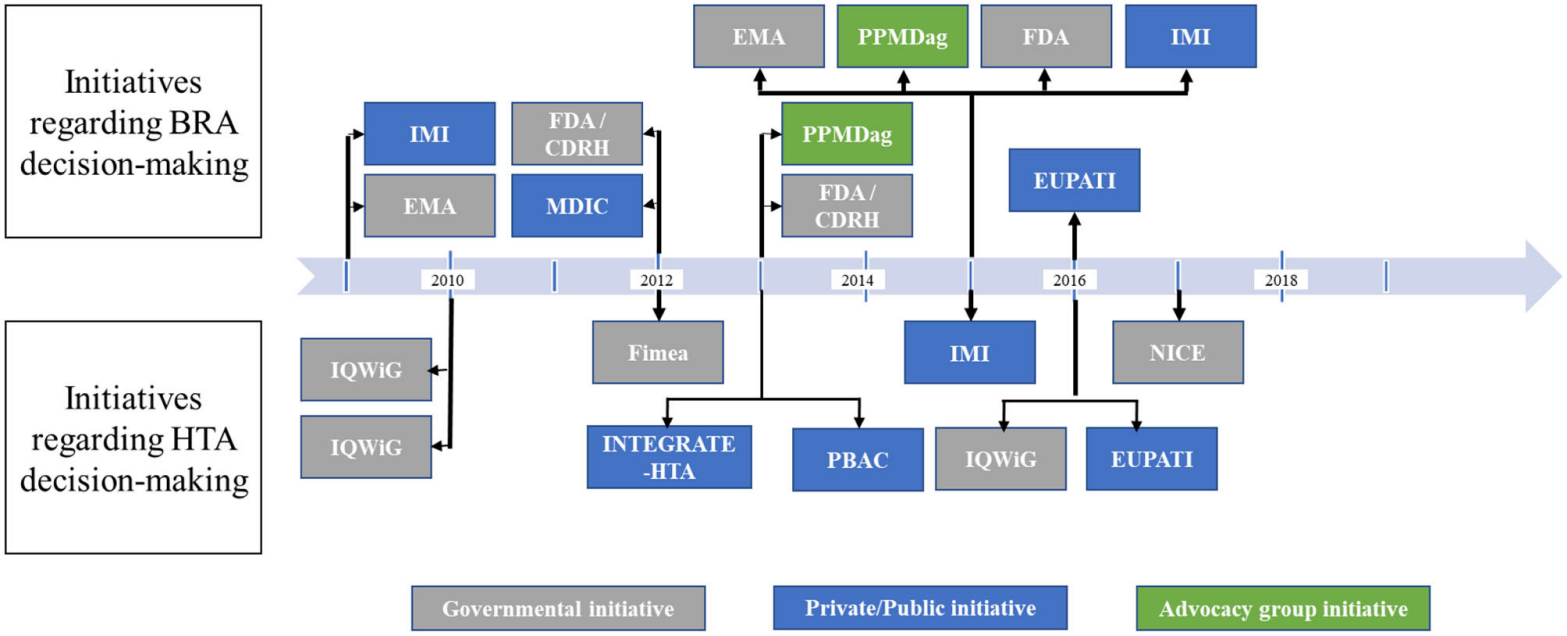
Chronological chart of main initiatives undertaken for integration of PPs in BRA and HTA decision-making. BRA, benefit–risk assessment; HTA, health technology assessment; IMI, Innovative Medicines Initiative; EMA, European Medicines Agency; FDA, US Food and Drug Administration; CDRH, Center for Devices and Radiological Health; MDIC, Medical device Innovation Consortium; PPMDag, Parent Project Muscular Dystrophy advocacy group; EUPATI, European Patients' Academy; IQWiG, Institute for Quality and Efficiency in Health Care; NICE, National Institute for Health and Care Excellence; Fimea, Finnish Medicines Agency; PBAC, Pharmaceutical Benefits Advisory Committee.

Attempts and initiatives worldwide, especially in the USA and in Europe, have begun to integrate PP elicitation, assessment, and valuation in major decision-making stages. Most recently, 20 noticeable endeavors, described in Figure 3, have been identified. Most of these initiatives (*n* = 13) were undertaken either at the European level (*n* = 8) or at the European country stage (*n* = 5) as has been the case in Germany, Finland, and the UK, while six initiatives were identified in the USA. The observable disparity is due to the difference in decision-making processes between European countries and the USA. In fact, market access pathway of new therapies in Europe is conditioned by a centralized two-step process: (1) marketing authorization granted at the European level by the European Medicines Agency (EMA) followed by (2) country-specific market access resulting to HTA, pricing, and reimbursement negotiations. However, in the USA, the centralized process is a one-step evaluation undertaken by the Food and Drug Administration (FDA) followed by decentralized negotiations undertaken with individual health insurance vendors. Moreover, only one initiative has been identified in Australia in the context of the HTA process (20).

#### Incorporation of PPs in BRAs

BRA is the process of determining whether the benefits of a treatment outweigh the risks, harms, and/or costs enough for regulatory approval by evaluating the efficacy, safety, and quality of the treatments (5, 6, 36, 40–43). In regulatory approval, decisions are based on clinical outcomes and endpoints valued by regulatory officials only (14, 42).

##### European attempts and initiatives

All the initiatives identified in Europe for incorporation of PPs in BRAs (*n* = 5) were undertaken at the European level (Table 3). The first initiative identified was undertaken by the EMA in 2009. The EMA in coordination with the Innovative Medicine Initiative (IMI) has launched the first European initiative for PP involvement in BRA decision making: the Pharmaco-epidemiological Research on Outcomes of Therapeutics by a European Consortium (PROTECT) project (41, 42). Even though the EMA has not issued any guidance related to implementation of PPs in marketing authorization decisions, the project has helped in defining the list of methods to be used for PP elicitation. Upon reviewal of methods and tools, the PROTECT project recommended the DCE as preferred tool for PP elicitation (41, 42). Moreover, within the 2013 EMA Patients' and Consumers' Organizations meeting, the EMA has conducted a pilot study among various groups of stakeholders, including patients, regulators, and healthcare providers, to outline methodologies for engaging patients and patient organizations in the regulatory decision-making process to generate, collect, and assess complementary PPs to inform these decisions (22). Results from this pilot study indicated that online MCDA surveys as well as swing weighting methods were the most feasible methods while revealing a great deal of heterogeneity in preferences (22).

**Table 3:** Initiatives for incorporation of PPs in BRA decision making.

**Institutional level**	**Scope**	**Institution**	**Year**	**Purpose of the initiative**	**References**
Governmental agencies	USA	FDA (CDRH)	2012	Pilot study (DCE eliciting PPs regarding a weight loss) to explore feasibility of PP studies and implementation of quantitative PPs in BRA	(4)
			2013–2017	Financial support for establishing CDER's Patient-Focused Drug Development initiativePublication of priority list of 20 disease areas that could benefit from the initiative	(5, 28)
		FDA	2015	Guidance on DMD and related disorders mentioning consideration of patient and caregiver risk tolerance in light of the life-threatening nature of the condition	(28)
	Europe	EMA	2009	Coordination of PROTECT collaborative project in partnership with IMINo specific guidance issued by the EMA	(41)
			2013–2015	EMA Patients' and Consumers' Organizations meeting conduction of a pilot study to assess feasibility and usefulness of systematically eliciting PPs for inclusion in BRA	(33, 42)
Private/public partnerships	Europe	IMI	2009	Launch of PROTECT collaborative project including two work packages dedicated to public/patient involvement in BRARecommendation of DCEs as preferred “utility survey technique”	(41, 42)
			2015	Launch of the 5th Call under IMI 2: Patient perspective elicitation on benefits and risks of medicinal products, from development through the entire life cycle, to inform the decision-making process by regulators and HTA bodies (PREFER project)	(42)
		EUPATI	2016	Patient training modules and guidance document development on PP elicitation and use in decision making (R&D, BRA, and HTA)	(10)
	USA	MDIC	2012	A framework to support FDA and industry in integration of PP in BRA of innovative medical devicesMDIC Methods Catalog: a general overview of available methods to quantify PP for BRA	(35)
Patient organizations and advocacy groups	USA	Parent Project Muscular Dystrophy Advocacy Group	2013	Development of the first patient-advocacy-initiated draft guidance for inclusion of PPs in regulatory decision making	(28)
			2015	PP pilot study that elicits preferences for a therapeutic agent that has demonstrated pulmonary benefit in a phase III clinical trial	(29)

*FDA, US Food and Drug Administration; EMA, European Medicines Agency; IMI, Innovative Medicines Initiative; EUPATI, European Patients' Academy; MDIC, Medical Device Innovation Consortium; CDRH, Center for Devices and Radiological Health; DMD, Duchenne muscular dystrophy; DCE, discrete choice experiment; BRA, benefit–risk assessment; HTA, health technology assessment; R&D, research and development; PP, patient preference*.

Furthermore, the IMI also launched some innovative initiatives regarding PP elicitation and incorporation. In July 2015, the IMI 2 Joint Undertaking established a 5-year proposal for PP elicitation to determine the valuation of risks and benefits for medicinal products throughout the products' life cycle to be integrated and weighted not only during BRAs but also within HTAs (33, 42). This project, called the Patient Preferences in Benefit–Risk Assessments during the Drug Life Cycle (PREFER), is co-led by 33 partners: 10 academic institutions from different European countries, 16 pharmaceutical companies from the USA and Europe, 4 national and international patient organizations, 1 HTA body, and 2 small- and medium-sized enterprises, all adding their experiences and perspectives to the project (12). The goal of this endeavor is the development of recommendations for PP inclusion throughout the entire medicinal product life cycle (3, 44). Lastly, in 2016, the European Patients' Academy (EUPATI) has also developed various training materials, documents, and workshops for patients to be involved in PP elicitation for inclusion in decision making (10).

##### US attempts and initiatives

No less than six initiatives have been identified in the USA: three projects funded by the FDA, two initiatives undertaken by patient organizations, and only one that was handled by a public/private partnership (Table 3). In 2012, the FDA's Safety and Innovation Act and the Prescription Drug User Fee Act V (PDUFA V) amendments (fiscal years 2013–2017) allowed for the founding of the Patient-Focused Drug Development initiative (5). This initiative was dedicated to promoting the generation, inclusion, and assessment of PPs with a more systematic and formal approach than ever before (5, 28). The FDA published a list of 20 disease areas, where this initiative's focus would be targeted for the first 3 years with an aim to elicit patients' experiences with their treatments, including benefits and risks as well as barriers to treatment access, and to inform the regulatory process (5, 28). In addition, the FDA also established the Patient Representative Program, which allows patients to partake in the BRA and regulatory discourse by having representatives included on committees and advisory boards involved in these processes (2).

In 2012, the US Center for Devices and Radiological Health (CDHR) launched the Patient Preference Initiative, establishing the framework for the generation, collection, and assessment of PPs for medical devices undergoing BRAs and in the regulatory approval process (5). A prime example of this initiative is the CDRH's use of DCE evaluation to reveal the PP for a weight loss medical device (4). The objective here was to determine the feasibility and best practice of obtaining PPs for regulatory decision making. Ultimately, this DCE was targeted to understanding the heterogeneity of the patient population to identify those willing to be on the treatment and those who were risk tolerant for market approval.

The Medical Device Innovation Consortium (MDIC), in the wake of the CDRH's 2012 pilot project on the significance of PPs and their integration in regulatory decision making, developed the MDIC Methods Catalog to complement and aid in the development of a framework to support the FDA and industry in the integration of PP in BRA of innovative medical devices (35). Not only does the Methods Catalog present a wide range of methods that may be applied for the elicitation and assessment of PP, it also does well to reiterate the potential and significance in the use and value of PP integration in the regulatory decision-making process (35).

In 2013, the Parent Project Muscular Dystrophy (PPMD), an advocacy group for Duchenne and Becker Muscular Dystrophies, was engaged by the FDA to draft the first patient-advocacy-initiated guidance on the elicitation, incorporation, and assessment of PPs. The PPMD submitted their first draft in June 2014. The FDA issued, the year after, its official guidance on DMD emphasizing that the FDA would be considering caregivers' and patients' preferences and risk tolerance in regulatory decisions (28, 37). No specification was mentioned regarding how PPs would be assessed and implemented.

The FDA's solicitation of the PPMD is a testament to its interest and growing dedication to include the patient voice and PPs in their regulatory processes as they continue to develop and publish guidelines. The administration has included patients, patient representatives, and caregivers in testimonial contributions to the regulatory process (37). Lastly, the FDA has also established and begun conducting public workshops to inform the development of patient-focused guidance (10).

#### Incorporation of PPs in HTAs

HTAs are centered around the evaluation of evidence and reimbursement decisions of new therapies. HTAs typically require clinical evidences as well as pharmacoeconomic studies, including cost-effectiveness analysis, budget impact analysis, and/or cost utility analysis (CUA) (13). By evaluating this evidence, HTA bodies are able to make their reimbursement decisions. However, patients have personal insight and differing views compared to HTA authorities as to what treatments they may prefer. HTA authorities may give varying priorities to other outcomes, and studies have confirmed discrepancies in assessments with multiple stakeholders (1, 2, 16). Traditionally, HTA processes have lacked the relative importance or preference of patients in relation to decision criteria and clinical endpoints (3, 23). Therefore, PPs may complement clinical evidence that HTA bodies evaluate by providing more than just quality of life on specific clinical outcomes, but instead on aspects of treatment that HTA authorities would not know otherwise, such as mode and frequency of administration—which is most convenient and the influence it has on patients to prefer one treatment over another (13, 26). To date, there has been little to no information regarding PP integration in HTA processes, and still, many HTA bodies worldwide recognize its importance but have yet to prioritize the methodologies and framework for including PP (3).

##### Attempts and initiatives in HTAs

With regard to the incorporation of PPs in the HTA decision process, nine attempts have been identified: three European private/public partnerships and six local governmental agencies initiatives (three in Germany, one in Finland, one in the UK, and one in Australia) (Table 4). In Europe, each country is likely to have its own HTA, and some countries may have regional HTAs. For instance, in Germany, the Institute for Quality and Efficiency in Health Care (IQWiG) conducts assessments of therapies based primarily on mortality, morbidity, and health-related quality of life related to new therapies. In 2010, a pilot project was initiated to elicit PPs for an antidepressant treatment via analytic hierarchy process (AHP) in patients with depression (8). The study also included healthcare provider preferences in order to identify potential deviances from patients' treatment preferences. IQWiG recognized that well-conducted AHP methods could aid the German HTA to determine patient-relevant outcomes and endpoints for incorporation and consideration in economic evaluations (8). Following this first pilot study, IQWiG launched a second study in 2010 to explore whether the use of the DCE method could inform health economic evaluations for chronic hepatitis C while making transparent PP elicitation (32). The German HTA is among the pioneers to apply such an exploratory study on a national basis (32). This study further informed IQWiG methodology of the Efficiency Frontier (EF), a multidimensional overall benefit concept serving as a framework for cost-effectiveness evaluations as well as indirectly for pricing and reimbursements of treatments (27).

**Table 4:** Initiatives for incorporation of PPs in HTA, pricing, and reimbursement decision making.

**Institutional level**	**Scope**	**Institution**	**Year**	**Purpose of the initiative**	**References**
Governmental agencies	Germany	IQWiG	2010	Pilot study: Use of PP studies (AHP) to weigh patient-relevant outcome within IQWiG appraisal in order to drive reimbursable prices for new medications	(8)
			2010	Pilot study: Use of PPs (DCE) to identify, weigh, and prioritize multiple patient-relevant outcomesInclusion of PPs in evidence-based decision making on the approval and pricing of innovations	(32)
			2016	Adaptation of the concept of the efficiency frontier to serve as a framework for the evaluation of cost-effectiveness and indirectly for the pricing and reimbursement of health technologies	(27)
	Finland	Fimea	2012	National recommendation consisting in a step-by-step guidance on how to conduct qualitative interviews (individual or focus groups) for integration of patients' voices into the HTA process of new pharmaceuticals	(9)
	Australia	PBAC	2013	Positive recommendation (exenatide 2 mg once weekly) following a DCE revealing potential health benefits from likely improved adherence	(20)
	UK	NICE	2017–2019	Funding a 2-year exploratory project to explore how to quantitatively capture and incorporate PPs in decision modeling as part of the HTA process (in collaboration with Myeloma UK)	(26)
Private/public partnerships	Europe	INTEGRATE-HTA	2013–2015	INTEGRATE-HTA work package 4 advocates for integration of social, cultural, ethical, legal, and organizational issues as well as patients' heterogeneity and preferences with effectiveness and cost-effectiveness in HTA	(45)
		IMI	2015	Launch of PREFER project: development of a systematic approach for considering the use of PPs across the medical treatment life cycle (time frame: 2016–2021)	(12)
		EUPATI	2016	Patient training modules and guidance documents development on PP election and use in decision making (R&D, BRA, and HTA)	(10)

*IQWiG, Institute for Quality and Efficiency in Health Care; NICE, National Institute for Health and Care Excellence; Fimea, Finnish Medicines Agency; PBAC, Pharmaceutical Benefits Advisory Committee; IMI, Innovative Medicines Initiative; EUPATI, European Patients' Academy; PP, patient preference; DCE, discreate-choice experiment; BRA, benefit–risk assessment; HTA, health technology assessment; R&D, research and development; AHP, analytic hierarchy process*.

Finland also launched a pilot study examining the potential for PP elicitation to be integrated in their HTA processes (9). The Finnish Medicines Agency (Fimea) applied this study to the area of diabetes seeking to further understand PP for insulin glargine (9). The study was based on interviews and concluded that they play a large role in qualitatively generating and assessing PPs. As a result of this pilot study, Fimea issued, in 2012, a national recommendation consisting of a step-by-step guidance on how to conduct qualitative interviews (individual or focus groups) for integration of patients' voices into the HTA process of new pharmaceuticals (9).

In the UK, the National Institute for Health and Care Excellence (NICE) is the HTA body responsible for reimbursement evaluation of treatments and has already shown interest and consulted on MCDA in their HTA decision-making process (26). Recently, Myeloma UK and NICE teamed together in a 2-year exploratory project to explore how best PPs could be quantitatively generated and assessed to inform the cost-effectiveness assessment as part of HTA decision making (26).

At the European level, the INTEGRATE-HTA project, co-founded by the European Union (EU), was dedicated to improving HTA methodologies to better fill in gaps that health authorities were missing in order to better build patient-centric solutions (45). The project which ended in December 2015 promoted inclusion of various backgrounds and stakeholders, including patients, and emphasized patient heterogeneity as an area of exploration (45). The INTEGRATE-HTA project also highlighted the current shortcomings of PP inclusion with relation to HTA and economic evaluations, such as cost-effectiveness (45). Moreover, the INTEGRATE-HTA project acknowledged that PP elicitation, assessment, and integration in decision making may also empower acceptability of health policy decisions and enhance the transparency of the decision-making processes (45). Lastly, the ongoing IMI PREFER project and EUPATI patients' training modules and guidance have also been working on tools and recommendations for use of PPs within the HTA process at the European level (3, 10, 12).

The Australian Pharmaceutical Benefits Advisory Committee (PBAC) has been considering PP information as part of a reimbursement dossier for exenatide 2 mg once weekly. The PBAC recommended the new therapy based on potential health benefits from likely improved adherence by a small number of high clinical need populations. The positive recommendation confirms the growing importance of providing patient perspectives and preferences during policy decision making (20).

## Discussion

### PP Definition and Elicitation

Although PPs have been studied and elicited for more than two decades, PP definition has been lastly proposed by the Center for Devices and Radiological Health (CDRH) under the umbrella of the FDA 2016. The CDRH has defined PP as “qualitative or quantitative assessments of the relative desirability or acceptability to patients of specified alternatives or choices among outcomes or other attributes that differ among alternative health interventions” (46); i.e., PPs refer to patient willingness to trade off between a set of good and bad outcomes or features related to different medical interventions. Thus, preferences may be expressed in terms of conveniences, inconveniences, burdens, and/or costs among other attributes (47). Although PP studies can cover a wide range of attributes and treatment features, treatment efficacy and safety turn out to be key components of medicinal product assessments as they are critical from a patient perspective and central in health policy decision making.

Historically, PPs have been rarely elicited and generated specifically for BRAs and HTAs (14). Increasingly, experts, governmental agencies, and prominent partnerships have begun to consider PPs as a substantial additional source for dossier submissions to health authorities considering the valuation and pricing and reimbursement for therapies (1, 13). The initiatives are undertaken at either the regional or local level. No global endeavor or consensus was yielded. Although all of the FDA, EMA, and local HTA agencies are exploring the use of PPs and PP studies in decision making (37, 43), no multistakeholder partnership has been undertaken to allow alignment on the use of PPs in decision making. Moreover, despite all these endeavors, PPs are not required to be included in marketing authorization applications and HTAs (3), and the role of such studies—their methodology and implementation—has not yet been agreed upon (6, 7, 28, 40).

Even though no consensus on how best to implement PPs in policy decision making was retrieved, elicitation methods for a collection of PPs were largely studied and used. Quantitative methods were favored to qualitative ones; however, the FDA acknowledged that qualitative and quantitative tools are two complementary approaches (5). While qualitative methods such as interviews, questionnaires, and focus groups tend to collect descriptive information from patients (3, 7, 8), these methods allow participation in small committees, which simplify and clarify tasks and facilitate discussion and knowledge sharing between experts and patients according to Marsh et al. (2). Moreover, these methods are often an essential step required for attribute selection and definition. Regarding quantitative PP elicitation tools identified, these methods constitute a large group of tools aiming at collecting preference measures while allowing statistical analysis (3, 7, 37), which may facilitate the introduction of formal evidence-based decision and adequate consideration when conducting a structured decision-making process (2, 5). These methods, summarized in Table 5, range from appealing easy-implementation tools such as scaling methods (ranking, rating, and visual analogue scale) with low cognitive burden (18) to more sophisticated ones such as choice-based methods eliciting preferences based on hypothetical scenarios (1). According to the EMA, choice-based methods (conjoint analysis, DCEs, and BWS) allow insight into which trade-offs result in a patient choosing a treatment, thus enabling regulators to evaluate these trade-offs (7). Furthermore, DCE, with its widespread use, has proven to be an important elicitation method for its ability to quantify the relative importance of different treatment characteristics through statistical analysis for incorporation within benefit–risk analyses as well as its ability to inform willingness to pay (WTP), which may be also used in cost–benefit economic evaluation (32, 34, 41). With regard to MCDA, this method is still at investigational stages in the field of policy decision making. The EMA and IQWiG have both experimented on the use of MCDA to elicit PPs in order to assist the medicinal product assessment process. The EMA has supported further investigation regarding the use of MCDA in BRAs, especially in cases where the benefit–risk may be marginal (42). According to Marsh et al., AHP, swing weighting, and paired comparison—all examples of MCDAs—may be used to support reimbursement and health technology decision making as they yield weighted outcomes and promote patient-centered decision making (2).

**Table 5:** Summary of the methods used to assess patient preferences.

**Method**	**Description of method**	**Strength**	**Weakness and limitations**	**References**
Ranking and rating	Direct scaling methods asking patient to rank or score attributes that distinguish treatment	• Feasibility of their implementation • Low cognitive burden	• Lack of direct explicit trade-offs between benefits and harms	(19, 43, 47)
Visual analogue scale	Raking method: Assign preference for a health state on a line anchored by perfect health and death	• Collection and valuation of several outcomes	• Use of ill-defined anchors which limit comparison between individuals • More valuable when used in combination with other methods	(16, 47)
Standard gamble	Choose either a gamble between perfect health and death or a certain but intermediate health state	• Estimation of quality-adjusted life-years (QALYs)	• Cognitively burdensome if several scenarios • Possibility of overestimation of patient's aversion to risk	(1, 19, 34, 41, 47)
Time trade-off	Choose either an intermediate health state for time *t* or perfect health for time *x* < *t*	• Estimation of QALYs • Assessment of risk preferences and minimum benefit	• Emotionally challenging for parents to consider their children having less years of life	(1, 19, 41, 47)
Discrete choice experiment	Choose between scenarios that describe a health state by different levels of attributes of that health state	• Valuation of hypothetical scenarios • Translation of preferences into utilities • Assessment of multiple attributes simultaneously • Ability to inform willingness to pay	• Require large sample sizes to produce statistically significant utilities	(1, 2, 13, 14, 21, 28, 30–32, 34, 40, 41, 43, 47, 48)
Best-worst scaling	Direct valuation of best and worst scenario or profile	• Less cognitively taxing on its participants	• Does not allow for “indifferent” choice	(13, 29, 31, 37, 43)
Multicriteria decision analysis	Direct consideration of an explicit set of criteria and their relative importance	• Decision based on several features simultaneously • Break down complex situations where many variables play a role in the decision-making process	• Potential cognitive burden • Requirement of a preliminary robust model	(2, 8, 27, 33, 42)
Analytic hierarchy process	Type of MCDA: Choose between multiple attributes or criteria in a pairwise compared manner	• Simplify complex decision making with multiple criteria, by reducing the trade-offs made at one time by presenting the choice as a pairwise comparison	• Valuation of limited number of outcomes • Potentially oversimplifying criteria and overlapping endpoints in complex pairwise hierarchies	(7, 8)
Swing weighting	Type of MCDA: First, patients rank the scale swings and afterwards allocate points that indicate the trade-off ratios	• Does not require econometric modeling: preferences are assumed to be directly captured with the elicitation task	• Potential cognitive burden requiring direct numerical assessment	(40)

### Importance of Incorporation and Consideration of PPs in Policy Decision Making

As patients are all unique, PP studies may be the key in understanding which treatment may be the best and for whom. Depending on their preferences, patients may vary greatly in their willingness to accept different degrees of risks to gain a minimum of benefit, so much so that there may even be substantial subgroups that would make very different decisions as to which therapy or product they would actually use (35, 47). Understanding PP and patients' values may further complement data presented to health authorities when deciding on market authorization as well as pricing and reimbursement, thus “providing care that is respectful of and responsive to individual patient preferences, needs, and values, and ensuring that patient values guide all clinical decisions” as defined by the Institute of Medicine (1, 16, 22, 46).

PP will only continue to gain importance as a patient-centric market becomes more preference sensitive. Preference-sensitive decisions will continue to arise when patients are presented with (a) more than one treatment option with no particular therapy being superior, (b) when therapy evidence and data are uncertain or variable, and/or (c) when patients' perspectives and preferences vary significantly from those of health authorities and decision makers (10, 29). Furthermore, PP inclusion may lead to increased accountability, reliability, and acceptance of health policy decisions (3, 8). Moreover, consideration of PPs may lead to improved patient compliance and adherence to therapy (49), further rendering the treatment more effective as the ability to guide patient treatment based on PP would result in decreased health costs and increased patient safety and benefits (30).

By anticipating PP and being able to understand the actual real-world benefits and harms of these therapies, complemented with the efficacy data provided by clinical evidence, health authorities may be able to make more efficient and effective decisions with regard to pricing and reimbursement as well as targeting of the optimal subgroups most likely to benefit—further enhancing the effectiveness of these therapies (45). In fact, the International Network of Agencies for Health Technology Assessment (INAHTA) conducted a survey in 2006 which revealed that including patients in these critical decision-making processes with health authorities expands the perspective and information available for the assessments and provides greater advice to them (8).

### Obstacles in Incorporation and Assessment of PPs

PP elicitation is becoming ever popular as patient-centric solutions are the new trend in healthcare. However, as with anything new and with any pioneers, obstacles can be identified. PP elicitation is still so new that the attempts and initiatives explored in this review still have not answered major questions. The only thing health authorities can agree on is that PPs are significant and can inform them on facets of patient experience and treatments they were not previously aware of.

However, critical questions remain as to who should be the source of PPs? How should patient be defined? Once a patient is defined, it is critical that the patient is educated on their role in PP elicitation and what exactly their input will be used for, which may be difficult (10, 24, 44). Those wishing to elicit PP should be wary of overwhelming the patient population by providing too much information but must educate the patient enough on the PP elicitation methodology for them to be able to engage in it. Scenarios presented via varying tools may be quite detailed, and patients must understand the hypothetical situations being presented to them, especially the attributes, the benefits, and the risks (44). Moreover, patients must understand the medical treatments being presented to them during the elicitation process (6). Some studies have expressed that there were doubts as to whether patients engaging in the studies were competent enough to be involved and to, ultimately, contribute to the decision-making process (10). If patients are inadequately educated and insufficiently involved, their participation will, ultimately, be futile (10). Of course, an inherent limitation to this is that in some studies where hypothetical situations are presented, real-life decisions could always be different, creating a bias (38). To offset this, other real-world trade-offs must be imitated and presented to the patients so as to reduce this bias as much as possible (38). Existing FDA recommendations explicitly state benefit—risk trade-off preference data should be patient centered with preferences elicited from “well-informed patients” (44).

Bias may also result from sources other than the patients themselves. Working with patient associations and advocacy groups has already been pursued in several attempts and initiatives, as mentioned with the FDA engaging patient associations to inform on DMD PP elicitation. While the collaboration resulted in guidance on the integration of PPs in the FDA's decision-making processes, it is not always the case for others. Engaging these types of groups may result in potential selection bias, yet they still remain critical in PP elicitation due to their ability to understand regulatory processes as well as their intimate knowledge and involvement in their disease areas (33).

There is still a need for reproducible, reliable, and generalizable methods to be efficiently employed for PP elicitation for the appropriate studies, stakeholders, and populations (44). Although this study has identified and detailed various methodologies, attempts, and initiatives, none are generalizable methods, and the guidelines developed cannot be clearly applicable to specific contexts—it is still unclear as to how, when, and where each or any of these methods or approaches should be employed (44).

Even though many relevant attempts have tried to elicit PP, it is not always very clear which guidelines the authors have followed and to which degree they have aligned with the guidelines if they have followed some (37). Moreover, while health authorities are doing well to recognize population heterogeneity, it is unclear how they will address this when healthcare solutions and treatments become increasingly patient centric and make difficult decisions in the best interest of all patients whose preferences are extremely heterogeneous (44). Whichever studies are ultimately deployed for PP elicitation must also meet consistent evidence standards in order to be incorporated and effectively evaluated. The FDA has issued some guidance calling for quality checks on stated-preference studies to ensure the results are sound and valid; however, the FDA has not detailed how the quality of these results should be measured (50).

Another aspect or potential obstacle for these studies that has not been thoroughly studied, yet merits further exploration, is the scope and expense of these studies. As patient-centric treatments and solutions will increasingly come to the market, the complexity of them and each additional attribute in the study of PP elicitation will be at a cost (45). The industry and decision makers will have to consider the cost of PP elicitation prior to engaging in any evaluation. Cost may also vary with the type of method employed to elicit PP (3). Ultimately, as with cost-effectiveness analyses for example, the argument can be made that with the application of the results of PP elicitation, further potential costs will be saved by making appropriate and informed decisions during BRAs and HTAs (45).

Reimbursement decisions are also political decisions, and incorporating PPs may not influence the final decision made by these health authorities (10). Patients and patient associations may want to pay as little as possible for any type of treatment, and with the right to vote and partake in these critical decision-making processes comes tremendous responsibility—hence, the dire importance for patients to be educated and competent enough to be integrated into these processes (10).

Time is also of the essence in some cases where treatments for extremely ill patients are on the line and being considered in major health authorities' decision-making processes. PP elicitation is itself a lengthy process. It takes time to define and identify patients and solicit them. It has been estimated that it takes from 6 months to 2 years to conduct a PP study, with recruitment of patients taking particularly longer than predicted (3). Guidelines should consider developing timeframes for conducting PP studies (11). Some have suggested that 3 months is an ample window of time to dedicate to PP study for decision-making inclusion and that time constraints should not be the driver as to whether systematic integration of PPs in regulatory decision making occurs (11). It is also not clear when in the decision-making processes of either BRAs or HTAs PP elicitation and PP studies should be conducted and submitted (3). There are currently no guidelines or, at least, requirements or deadlines for the incorporation of PP studies; these studies are simply accepted as supplementary to the dossier submission of these decision-making processes (3).

## Strengths and Limitations of This Review

To the authors' knowledge, this is the first review summarizing existing PP studies informing treatment preferences for health policy decision making as well as relating attempts and initiatives for inclusion of these PPs in BRAs, HTAs, and pricing and reimbursement decisions. The work also does well to define and categorize the various PP elicitation tools.

When results of the review are interpreted, limitations should be considered. Despite the willingness of investigating the implementation of PPs in policy decision making, the authors did not review HTA and BRA reports to directly look for utilization and consideration of PPs. Another limitation is the restriction of the search to only the PubMed database and to only English publications, which may have resulted in unaccounted for publications relevant to the search objectives, such as country-specific guidance in native languages for PP inclusion in decision-making processes. Moreover, as the concept of PPs and the official definition are very recent, the authors had to consider broad search terms to be sure to cover all the research covering PP notion.

## Conclusion

The importance of PPs is not negligible, particularly in providing additional evidence on drug efficacy, in promoting transparency and legitimacy for the patients as well as patient welfare and compliance by meeting their needs and expectations, and in increasing market authorization acceptance publicly.

Nowadays, PP elicitation tools are largely understood, and their use is better performed by researchers and experts. However, despite the efforts identified and the initiatives undertaken, the pace of progress remains slow. Evidence of proper use of these data in policy decision making is lacking as PPs remain poorly implemented. Moreover, important questions are still to be resolved: What is an appropriate structured approach to implement PPs within the approval of medical products? What level of validity, representativeness, and robustness is necessary? How will these PP approaches satisfy the needs of the different stakeholders, specifically regulatory, HTA, and reimbursement bodies, and feed into their existing decision-making processes? Further researches are required. Guidelines and recommendations formalizing PPs are needed to improve and empower integration of PP in BRA and HTA.

## Author Contributions

All authors contributed to the article and approved the submitted version.

## Conflict of Interest

MT, CF, and SA are affiliated to Aix Marseille University. They are also consultants for Creativ-Ceutical, a consulting company advising private and public organizations. This research is part of LC Ph.D. program at Aix Marseille University. The remaining authors declare that the research was conducted in the absence of any commercial or financial relationships could be construed as a potential conflict of interest.
